# Safety and immunogenicity of an inactivated recombinant Newcastle disease virus vaccine expressing SARS-CoV-2 spike: Interim results of a randomised, placebo-controlled, phase 1 trial

**DOI:** 10.1016/j.eclinm.2022.101323

**Published:** 2022-03-08

**Authors:** Punnee Pitisuttithum, Viravarn Luvira, Saranath Lawpoolsri, Sant Muangnoicharoen, Supitcha Kamolratanakul, Chaisith Sivakorn, Piengthong Narakorn, Somchaiya Surichan, Sumalee Prangpratanporn, Suttida Puksuriwong, Steven Lamola, Laina D. Mercer, Rama Raghunandan, Weina Sun, Yonghong Liu, Juan Manuel Carreño, Rami Scharf, Weerapong Phumratanaprapin, Fatima Amanat, Luc Gagnon, Ching-Lin Hsieh, Ruangchai Kaweepornpoj, Sarwat Khan, Manjari Lal, Stephen McCroskery, Jason McLellan, Ignacio Mena, Marcia Meseck, Benjaluck Phonrat, Yupa Sabmee, Ratsamikorn Singchareon, Stefan Slamanig, Nava Suthepakul, Johnstone Tcheou, Narumon Thantamnu, Sompone Theerasurakarn, Steven Tran, Thanakrit Vilasmongkolchai, Jessica A White, Nina Bhardwaj, Adolfo Garcia-Sastre, Peter Palese, Florian Krammer, Kittisak Poopipatpol, Ponthip Wirachwong, Richard Hjorth, Bruce L Innis

**Affiliations:** a Faculty of Tropical Medicine, Mahidol University, 420/6 Ratchawithi Road, Ratchathewi, Bangkok 10400, Thailand; b The Government Pharmaceutical Organization, 75/1 Rama VI Road, Ratchathewi, Bangkok 10400, Thailand; c PATH, 2201 Westlake Avenue, Suite 200, Seattle, WA 98121, USA; d Department of Microbiology, Icahn School of Medicine at Mount Sinai, 1 Gustave L. Levy Pl, New York, NY 10029, USA; e Nexelis, 525 Bd Cartier O, Laval, QC H7V 3S8, Canada; f College of Natural Sciences, The University of Texas at Austin, 120 Inner Campus Dr Stop G2500, Austin, TX 78712, USA; g Global Health and Emerging Pathogens Institute, Icahn School of Medicine at Mount Sinai, 1 Gustave L. Levy Pl, New York, NY 10029, USA; h The Tisch Cancer Institute, Icahn School of Medicine at Mount Sinai, 1 Gustave L. Levy Pl, New York, NY 10029, USA; i Department of Medicine, Icahn School of Medicine at Mount Sinai, 1 Gustave L. Levy Pl, New York, NY 10029, USA; j Department of Pathology, Icahn School of Medicine at Mount Sinai, 1 Gustave L. Levy Pl, New York, NY 10029, USA

## Abstract

**Background:**

Production of affordable coronavirus disease 2019 (COVID-19) vaccines in low- and middle-income countries is needed. NDV-HXP-S is an inactivated egg-based recombinant Newcastle disease virus vaccine expressing the spike (S) protein of severe acute respiratory syndrome coronavirus 2 (SARS-CoV-2). It's being developed by public sector manufacturers in Thailand, Vietnam, and Brazil; herein are initial results from Thailand.

**Methods:**

This phase 1 stage of a randomised, dose-escalation, observer-blind, placebo-controlled, phase 1/2 trial was conducted at the Vaccine Trial Centre, Mahidol University (Bangkok). Healthy males and non-pregnant females, aged 18–59 years and negative for SARS-CoV-2 antibodies, were eligible. Participants were randomised to receive one of six treatments by intramuscular injection twice, 28 days apart: 1 µg, 1 µg+CpG1018 (a toll-like receptor 9 agonist), 3 µg, 3 µg+CpG1018, 10 µg, or placebo. Participants and personnel assessing outcomes were masked to treatment. The primary outcomes were solicited and spontaneously reported adverse events (AEs) during 7 and 28 days after each vaccination, respectively. Secondary outcomes were immunogenicity measures (anti-S IgG and pseudotyped virus neutralisation). An interim analysis assessed safety at day 57 in treatment-exposed individuals and immunogenicity through day 43 per protocol. ClinicalTrials.gov (NCT04764422).

**Findings:**

Between March 20 and April 23, 2021, 377 individuals were screened and 210 were enroled (35 per group); all received dose one; five missed dose two. The most common solicited AEs among vaccinees, all predominantly mild, were injection site pain (<63%), fatigue (<35%), headache (<32%), and myalgia (<32%). The proportion reporting a vaccine-related AE ranged from 5·7% to 17·1% among vaccine groups and was 2·9% in controls; there was no vaccine-related serious adverse event. The 10 µg formulation's immunogenicity ranked best, followed by 3 µg+CpG1018, 3 µg, 1 µg+CpG1018, and 1 µg formulations. On day 43, the geometric mean concentrations of 50% neutralising antibody ranged from 122·23 international units per mL (IU/mL; 1 µg, 95% confidence interval (CI) 86·40–172·91) to 474·35 IU/mL (10 µg, 95% CI 320·90–701·19), with 93·9% to 100% of vaccine groups attaining *a* ≥ 4-fold increase over baseline.

**Interpretation:**

NDV-HXP-S had an acceptable safety profile and potent immunogenicity. The 3 µg and 3 µg+CpG1018 formulations advanced to phase 2.

**Funding:**

National Vaccine Institute (Thailand), National Research Council (Thailand), Bill & Melinda Gates Foundation, National Institutes of Health (USA).

**Research in context**

**Evidence before this study**

We aspired to repurpose egg-based influenza vaccine manufacturing facilities in low- and middle-income countries (LMICs) to make an affordable coronavirus disease 2019 (COVID-19) vaccine based on a recombinant Newcastle disease virus (NDV) expressing the severe acute respiratory syndrome coronavirus 2 (SARS-CoV-2) spike (S) protein, which replicates well in chicken eggs. We searched PubMed for research articles published between database inception and July 25, 2021, using the terms “inactivated” and “vaccine” and “Newcastle disease virus” and “COVID-19″ with no language restriction. A single report provided pre-clinical evidence suggesting this approach was feasible. A subsequent report posted to bioRxiv 2021 presented additional evidence that a recombinant NDV vaccine expressing a well-stabilized S protein construct was highly protective in the hamster model of disease.

**Added value of this study**

This study reports encouraging results from the first use of the NDV-HXP-S COVID-19 vaccine candidate in human subjects. In a trial conducted in Thailand, using domestically produced vaccine, all five vaccine formulations tested had an acceptable safety profile relative to placebo, and elicited dose-dependant levels of vaccine-homologous anti-S IgG and pseudotyped virus neutralising antibodies that exceeded those in human convalescent sera, suggesting the vaccine candidate may be highly protective.

**Implications of all the available evidence**

These results support that the NDV-HXP-S COVID-19 vaccine has great promise, as it can be manufactured in LMICs using a simple, inexpensive, highly scalable process. Additional studies of this vaccine to confirm its optimal formulation and its acceptable safety and immunogenicity relative to a comparator vaccine with demonstrated efficacy against COVID-19, could support its authorization for emergency use in 2022. Manufacturers in Vietnam and Brazil are also developing this product, which could advance more equitable global access to COVID-19 vaccines.

Alt-text: Unlabelled box

## Introduction

There remains a shocking imbalance in the global distribution of coronavirus disease 2019 (COVID-19) vaccines.^1^ To achieve control of the COVID-19 pandemic in low- and middle-income countries (LMICs) where most of the global population resides, there must be a great increase in sustainable supply of affordable vaccines. The manufacturing capacity for egg-based inactivated influenza vaccines (IIV) is among the largest in the world; these facilities, some in middle-income countries and operating for less than six months per year, use locally produced embryonated eggs to make more than a billion doses annually of affordable human vaccines.^2^ To enable these manufacturers to respond to the COVID-19 pandemic, we developed a COVID-19 vaccine for production in eggs, based on a recombinant Newcastle disease virus (NDV) expressing the ectodomain of a novel membrane-anchored, prefusion-stabilized severe acute respiratory syndrome coronavirus 2 (SARS-CoV-2) spike (S) protein construct, wherein virions are purified and inactivated (NDV-HXP-S).^3^, ^4^, ^5^

From September to November 2020, manufacturers in Thailand, Vietnam, and Brazil modified their IIV manufacturing process to optimize production of beta-propiolactone (BPL)-inactivated NDV-HXP-S, achieving high yields at pilot scale; the result was three similar processes. A preclinical evaluation of their vaccine candidates, formulated with and without CpG1018, a toll-like receptor 9 (TLR-9) agonist adjuvant (Dynavax Technologies)^6^ confirmed that they were highly immunogenic and protective in hamsters^5^ with no sign of toxicity in rats at the maximum human doses planned for evaluation (3 µg S protein+1·5 mg CpG1018; 10 µg S protein). These results enabled all three manufacturers to initiate clinical development of their vaccine candidates. Herein, we report interim safety and immunogenicity data generated in the phase 1 portion of a phase 1/2 clinical trial evaluating the NDV-HXP-S vaccine candidate developed by The Government Pharmaceutical Organization of Thailand (GPO). The clinical development program for the NDV-HXP-S vaccine candidate in Thailand began in March 2021, shortly after the Government procured its first COVID-19 vaccines from Sinovac and AstraZeneca; these products were authorised for emergency use by Thailand's Food and Drug Administration and administered to health care personnel, older adults, and other high-risk groups. Our aim is to attain emergency use authorisation for the NDV-HXP-S vaccine candidate in 2022 to supply a domestically produced, affordable vaccine for COVID-19 prevention and control. Our results herein provide the first evidence in humans that a recombinant NDV expressing a six-proline prefusion-stabilized S protein offers a unique platform for affordable manufacturing of a well-tolerated and highly immunogenic COVID-19 vaccine.

## Methods

### Study design and participants

The phase 1 segment of a randomised, observer-blind, placebo-controlled, phase 1/2 trial, to assess the reactogenicity, safety, and immunogenicity of five formulations of the NDV-HXP-S vaccine candidate with the objective to advance two to phase 2, was performed at the Vaccine Trial Centre, Faculty of Tropical Medicine, Mahidol University (Bangkok, Thailand). Participants were recruited from individuals known to the Centre from prior studies plus their family and friends, and through advertisements. Healthy males and non-pregnant females, 18–59 years of age with body mass index 17 to 40 kg/m^2^, and negative for hepatitis B surface antigen and SARS-CoV-2, HIV, and hepatitis C antibodies were eligible to participate. A negative urinary pregnancy test was required of women having reproductive capacity prior to administration of each study vaccine dose. Complete eligibility criteria are described in the trial protocol provided in the supplementary material.

Written informed consent was obtained from all participants. The trial complied with the Declaration of Helsinki and Good Clinical Practice. This study was approved by the Ethics Committee of the Faculty of Tropical Medicine, Mahidol University (TMEC 21–005) and authorized by the Thailand Food and Drug Administration (FDA-21–018). The reporting of this trial complies with the current version of the Consolidated Standards of Reporting Trials (CONSORT) 2010 Statement.

### Randomisation and masking

Enroled subjects were randomly assigned in sequence to one of 6 equal groups (vaccine containing 1 µg S, 1 µg *S* + 1·5 mg CpG1018 adjuvant, 3 µg S, 3 µg *S* + 1·5 mg CpG1018 adjuvant, 10 µg S, or saline placebo). Subjects were enroled in stages, each including active treatment and placebo groups, using a computer-generated randomisation sequence prepared by a statistician otherwise uninvolved in the study; an unblinded pharmacist team dispensed each treatment according to the randomisation sequence. The first 18 subjects (sentinel cohort) were enroled to three sequential sentinel groups; 3:1, 1 µg and placebo; 3:3:1, 3 µg or 1 µg+CpG1018 and placebo; and 3:3:1, 10 µg or 3 µg+CpG1018 and placebo, After safety data were reviewed for the sentinel groups, the next 192 subjects were randomised in five dose-cohorts; 32:6, 1 µg and placebo; 32:6, 3 µg and placebo; 32:6, 1 µg+CpG1018 and placebo; 32:7, 10 µg and placebo; and 32:7, 3 µg+CpG1018 and placebo. All participants and personnel other than the unmasked pharmacy team and vaccinators were masked to treatment.

### Procedures

The recombinant NDV-HXP-S vaccine was manufactured according to current Good Manufacturing Practice by the GPO in their Influenza Vaccine Plant (Saraburi, Thailand) using locally procured embryonated eggs inoculated with a master virus seed made and extensively tested for adventitious agents by the Icahn School of Medicine at Mount Sinai (New York, USA). After incubation for 72 h at 37 °C, eggs were chilled overnight at 4 °C, then the allantoic fluids were harvested, clarified, and concentrated. Recombinant virus particles were purified from the concentrated harvest by two sequential continuous flow sucrose gradient centrifugations, diafiltered against phosphate-buffered saline (PBS), inactivated by treatment with 1:4000 BPL for 24 h at 4 °C, and 0.2 µm filter-sterilized. Vaccine potency (i.e., amount of HXP-S antigen per dose) was measured by direct enzyme-linked immunosorbent assay (ELISA) using a human monoclonal antibody (CR3022^7^) to SARS-CoV-2 S1 glycoprotein (LakePharma Inc) and an NDV-HXP-S standard that had been calibrated to a purified HXP-S reference^8^ by sodium dodecyl sulphate polyacrylamide gel electrophoresis (SDS-PAGE) densitometry.

Unmasked staff administered study treatments by intramuscular injection of 0·5 mL on study days 1 and 29. Blood samples were drawn and clinical assessments were done for safety and immunogenicity endpoints before vaccination on days 1 (dose one), 8, 29 (dose two), 36, and 43; a clinical assessment for safety only on day 57 was the last timepoint considered for the interim analysis of the phase 1 cohort, although there will be additional immunogenicity and safety assessments on study day 197. Subjects were observed in the clinic for 30 min after each vaccination and were asked to record any adverse events using paper diary cards during the 7-days after each vaccination. Subjects randomly allocated to a cell-mediated immunity subset (*N* = 12 per 10 µg, 3 µg+CpG1018, and placebo groups) had additional blood collected on days 1 and 43 for isolation of peripheral blood mononuclear cells (PBMCs); these were stored in liquid nitrogen until analysed.

Solicited injection site reactions (pain, tenderness, swelling, induration, erythema) and systemic symptoms (headache, fatigue, malaise, myalgia, arthralgia, nausea, vomiting, and fever defined as oral temperature ≥ 38 °C) were recorded by subjects in a diary card that included intensity, then reported by the investigators; these events were not assessed for causality. Subjects also recorded spontaneously reported adverse events (AEs) for 28 days; the investigator reported these after grading them for intensity and categorizing them as serious or not. The investigator also identified the following AEs of special interest: potential immune-mediated medical conditions and AEs associated with COVID-19. Intensity of AEs was graded 1–4 as follows: 1 or mild (minimal interference with daily activities), 2 or moderate (interferes with but does not prevent daily activities), 3 or severe (prevents daily activities, intervention required), and 4 or very severe (medical intervention required to prevent disability or death). Investigators assessed spontaneously reported adverse events for causality (related to vaccination or not). AEs were graded according to U.S. Department of Health and Human Services severity grading tables (Food and Drug Administration, Centre for Biologics Evaluation and Research [September 2007] and National Institutes of Health, Division of AIDS [version 2.1, July 2017]). A protocol safety review committee regularly reviewed blinded safety data; a Data Safety Monitoring Board monitored unblinded safety data and recommended two formulations for advancing to phase 2.

We measured total anti-SARS-CoV-2 S IgG using a validated indirect ELISA at Nexelis (Laval, Canada). Purified recombinant SARS-CoV-2 pre-fusion S (Nexelis) at 1 µg/ml in PBS (Wisent Bioproducts) was adsorbed to 96 well Nunc Maxisorb microplates (Thermo Fischer Scientific) and blocked with 5% skim milk in PBS, containing 0·05% Tween 20. Serial dilutions of test samples and the assay standard plus controls were added in the plates and incubated for 60 min at room temperature (15–30 °C). After washing, horseradish peroxidase (HRP) enzyme-conjugated goat anti-human IgG-Fc (Jackson ImmunoResearch Laboratories) was added for 60 min at room temperature (15–30 °C), then washed. Bound secondary antibody was reacted with 3,3′,5,5′-tetramethylbenzidine (TMB) ELISA peroxidase substrate (Bio-Rad Laboratories) and incubated for 30 min at room temperature (15–30 °C) before the reaction was stopped with 2 N H_2_SO_4_. Plates were read at 450 nm with a correction at 620 nm to assess the level of anti-S IgG bound in the microtiter plate. A reference standard on each plate determined the quantity of anti-S IgG in arbitrary units (ELU/mL). Concentrations were transformed to binding antibody units per mL (BAU/mL), based on the World Health Organization (WHO) International Standard for anti-SARS-CoV-2 immunoglobulin,^9^ using a conversion factor determined during assay validation (1/7·9815). The assay's cut-off and lower limit of quantification (LLOQ) was 6·3 BAU/mL.

We measured serum neutralising activity against the Wuhan-Hu-1 strain of SARS-CoV-2 in a validated pseudotyped virus neutralisation assay (PNA) that assessed particle entry-inhibition.^10^ Briefly, pseudotyped virus particles containing a luciferase reporter for detection were made from a modified vesicular stomatitis virus (VSVΔG) backbone expressing the full-length S protein of SARS-CoV-2 (MN908947, Wuhan-Hu-1) from which the last 19 amino acids of the cytoplasmic tail were removed.^11^ Seven two-fold serial dilutions of heat-inactivated serum samples were prepared in 96-well round-bottom transfer plates (Corning). Pseudotyped virus was added to the serum dilutions at a target working dilution and incubated at 37 °C with 5% CO_2_ for 60 ± 5 min. Serum-virus complexes were then transferred onto 96 well white flat-bottom plates (Corning), previously seeded overnight with Vero E6 cells (Nexelis) and incubated at 37 °C and 5% CO_2_ for 20 ± 2 h. Following this incubation, luciferase substrate from ONE Glo™ Ex luciferase assay system (Promega) was added to the cells. Plates were then read on a SpectraMax® i3x plate reader (Molecular Devices) to quantify relative luminescence units, inversely proportional to the level of neutralising antibodies present in the serum. The neutralising titre of a serum sample was calculated as the reciprocal serum dilution corresponding to the 50% neutralisation antibody titre (NT_50_) for that sample; the NT_50_ titres were transformed to international units per mL (IU/mL), based on the WHO international standard for anti-SARS-CoV-2 immunoglobulin, using a conversion factor determined during assay validation (1/1·872). The assay's cut-off and LLOQ were 5·3 IU/mL (10 as NT_50_) and 5·9 IU/mL, respectively. To benchmark vaccine immunogenicity assessed in BAU/mL and IU/mL, we used a panel of human convalescent serum samples (HCS) collected 14 days after symptom onset from consecutive cases of mild to moderate COVID-19 illness among health care personnel seen as outpatients in Quebec, Canada during mid-2020. We also calculated 80% neutralisation titres (NT_80_); nevertheless, as the PNA was not validated for this measurement, these results are not presented. We used the same PNA assay to measure NT_50_ (reported as titres) against pseudotyped virus particles generated for SARS-CoV-2 variants of concern B.1.315,^12^ P.1, and B.1.617.2.^13^ In the absence of positive controls for the variant strains of SARS-CoV-2, we used control sera for the Wuhan-Hu-1 strain.

To assess cellular immunity, we quantified interferon-γ (IFN-γ) and interleukin-5 (IL-5) producing cells in PBMCs stimulated with SARS-CoV-2 S peptide pools (vial 1, 158 overlapping peptides; vial 2, 157 overlapping peptides; JPT Peptide) using a human IFN-γ/IL-5 double-colour enzyme-linked immunosorbent spot (ELISpot) kit (Cellular Technologies) in a qualified assay. Briefly, activated 96-well plates were coated with anti-human IFN-γ/IL-5 capture antibodies at 2–8 °C. Following overnight (> 16 h) coating, plates were washed with PBS, and stimulation media containing SARS-CoV-2 S peptide pool 1 or peptide pool 2 or control media was added to wells, followed by the addition of PBMCs at 2 × 10^5^ cells/well. After an approximately 44-hour incubation at 37 °C ± 1 °C with 5% CO_2_, plates were washed to remove cells from the wells. Anti-human IFN-γ/IL-5 detection solution (containing anti-human IFN-γ fluorescein isothiocyanate [FITC] and anti-human IL-5 [biotin] detection antibodies) was then added to the wells and incubated at room temperature (15–30 °C) for 2 h ± 10 min to detect IFN-γ and IL-5 cytokine captured on the bottom of the well. Plates were washed, followed by the addition of a tertiary solution (containing FITC—HRP and streptavidin-alkaline phosphatase). Following incubation with the tertiary solution, plates were washed, and blue and red developer solutions were added in sequence (with washes in between), resulting in the appearance of blue (for IL-5) and red (for IFN-γ) spot forming units (SFUs) in proportion to T cell activity. SFUs were counted by an ImmunoSpot CTL analyser (using CTL ImmunoCapture Software (v7·0·14·0) and CTL ImmunoSpot Professional DC analyser (v7·0·28·2)). Readouts (one per peptide pool for IFN-γ, one per peptide pool for IL-5) were expressed as number of SFU/10^6^ cells and combined as a ratio. The assay's LLOQ for IFN-γ was 109 SFU/10^6^ cells and for IL-5 was 43 SFU/10^6^ cells.

### Outcome

The primary outcomes were frequency and intensity of solicited injection site and systemic AEs during 7 days after vaccination; frequency, intensity, and relatedness of clinically significant haematological and biochemical measurements at 7 days after each vaccination; frequency, intensity, and relatedness of unsolicited AEs during 28 days after each vaccination; and occurrence of medically-attended AEs, serious AEs, and AEs of special interest during the interim analysis period of 57 days after-first vaccination. The secondary immunogenicity outcomes were anti-S IgG and NT_50_ against Wuhan-Hu-1 strain SARS-CoV-2 pseudotyped virus assessed on days 29 and 43 and expressed as geometric mean titre (GMT) or concentration (GMCs, BAU/mL for ELISA, or IU/mL for PNA), geometric mean fold rise (GMFR) from baseline, and percentage of subjects with ≥4-fold increase and ≥10-fold increase from baseline. The exploratory immunogenicity outcomes were cellular immunity to SARS-CoV-2 S protein, measured as the ratio of IFN-γ/IL-5 expressing cells on days 1 and 43 in a random subset of subjects receiving two vaccine formulations (10 µg or 3 µg+CpG1018) or placebo. We also assessed NT_50_ GMTs and the percentage of subjects seronegative for anti-S IgG on day 1 and with a NT_50_ titre ≥ 40 on day 43 against vaccine heterologous SARS-CoV-2 pseudotyped virus variants of concern B.1.351 and P.1 for all vaccine formulations and B.1.617.2 for the 3 µg formulation only.

### Statistical analyses

This phase 1/2 study (ClinicalTrial.gov NCT04764422) has a two-part selection design with group elimination after the interim analysis. In the first part, 35 subjects per group were randomised across 5 candidate vaccine formulations and a placebo group for a total of 210 subjects. After the interim analysis, two candidates were selected to advance, at which time 250 additional subjects were to be randomised 2:2:1 to the two selected candidate groups and the placebo, respectively. The phase 1 portion of the study included 35 subjects per group, affording greater than 80% probability to observe at least one serious or severe AE if the true event rate is 5% or more.

All statistical tests were two-sided with a significance level of 0·05. All statistical analyses were performed using SAS version 9·4. All safety assessments took place in the treatment-exposed population, according to the treatment received. All treatment group percentages were supplemented with two-sided 95% confidence intervals (CIs) computed via the Clopper-Pearson method. The analysis of immunogenicity was performed in the per protocol population, which excluded subjects with protocol deviations that would affect the assessment. Immunogenicity data were descriptively analysed. Geometric mean antibody responses were reported by treatment and time point, accompanied by 95% CIs. The analysis of geometric means excluded subjects who were seropositive at baseline (defined by anti-S IgG >LLOQ as measured by ELISA). GMFRs were calculated relative to baseline using the log difference of the paired samples, with corresponding CIs computed via the *t*-distribution, utilizing the antilog transformation to present the ratio. The proportions of subjects with GMFRs of NT_50_ ≥4 and ≥10 from baseline were summarized with two-sided 95% confidence intervals computed via the Clopper-Pearson method. The analysis of immunogenicity relative to baseline included baseline seropositive subjects.

### Role of funding source

The funders of the study had no role in data collection, data analysis, or writing of the statistical report. GPO was the clinical trial sponsor and approved the study protocol. GPO employees contributed as authors by preparing the investigational vaccine, interpreting data, and writing this report. All authors had full access to all the data in the study and accept responsibility for the decision to submit for publication.

## Results

**Figure 1:**
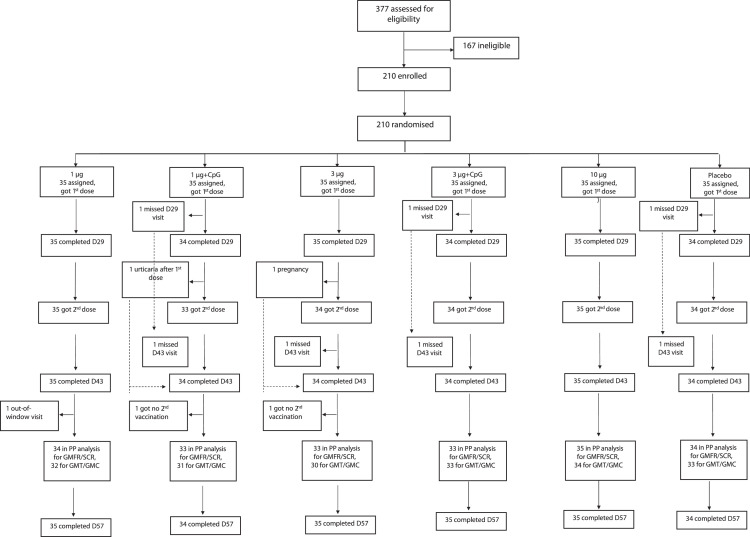
Trial profile.

Between March 22 and April 23, 2021, 210 healthy adults were enroled and assigned to one of six treatment groups. All received a first dose of vaccine or placebo; two subjects were excluded from receipt of a second dose (one became pregnant, one developed mild urticaria within 30 min after dose one); three other subjects missed the day 29 visit and got no second dose (Figure 1 identifies their group assignments). The baseline characteristics are shown by treatment group in Table 1; the exposed population was 61% female, had a median age of 36 years (inter-quartile range [IQR] 28–43) and a median body mass index of 24·07 (IQR 21·30–26·72).

**Table 1:** Baseline characteristics of the exposed population.

	1 µg S(*N* = 35)	1 µg S+CpG(*N* = 35)	3 µg S(*N* = 35)	3 µg S+CpG(*N* = 35)	10 µg S(*N* = 35)	Placebo(*N* = 35)
Age, years	33·0 (26·0–39·0) [18, 53]	39·0 (32·0–45·0) [20, 55]	37·0 (29·0–49·0) [18, 56]	34·0 (25·0–44·0) [19, 58]	37·0 (31·0–42·0) [19, 57]	32·0 (27·0–42·0) [19, 52]
Sex						
Male	14 (40·0%)	14 (40·0%)	7 (20·0%)	15 (42·9%)	18 (51·4%)	14 (40·0%)
Female	21 (60·0%)	21 (60·0%)	28 (80·0%)	20 (57·1%)	17 (48·6%)	21 (60·0%)
Ethnicity						
Asian	35 (100%)	35 (100%)	35 (100%)	35 (100%)	35 (100%)	35 (100%)
Body mass index	24·59 (20·76–27·85) [17·19, 35·06]	24·85 (21·42–26·33) [17·19, 30·06]	23·95 (21·23–27·96) [18·71, 33·73]	23·95 (21·70–25·92) [17·50, 31·79]	24·52 (21·26–27·68) [17·88, 35·06]	23·12 (21·72–27·22) [17·85, 32·78]

Data are median (q1-q3) and [min, max] or n (%).

All five formulations of NDV-HXP-S were well tolerated with no dose limiting reactogenicity (Table 2). Most solicited injection site and systemic reactogenicity during 7 days after each vaccination was mild and transient with no apparent difference between dose one and two. The most common injection site symptoms (Table 2) were pain and tenderness; these were most frequent at the highest dose. The most common systemic symptoms (Table 2) were fatigue, headache, and myalgia, generally in less than one-third of subjects. Fever was uncommon. AEs occurring during 28 days after vaccination (Table 3) and judged by the investigator to be treatment-related were infrequent (< 15%) and there was no treatment-related serious AE, nor any AE of special interest reported during the 57 day assessment period. Haematology and serum chemistry laboratory readouts were assessed on day 8 following each vaccination; there was no clinically notable finding relative to baseline assessment. The Data Safety Monitoring Board expressed no safety concern.

**Table 2:** Solicited AEs during 7 days after vaccination.

		1 µg S(*N* = 35)	1 µg S+CpG(*N* = 35)	3 µg S(*N* = 35)	3 µg S+CpG(*N* = 35)	10 µg S(*N* = 35)	Placebo(*N* = 35)
		n (%)(95% CI)	n (%)(95% CI)	n (%)(95% CI)	n (%)(95% CI)	n (%)(95% CI)	n (%)(95% CI)
Any injection site AE	Dose 1	8 (22·9%) (10·4–40·1)	13 (37·1%) (21·5–55·1)	14 (40·0%) (23·9–57·9)	20 (57·1%) (39·4–73·7)	23 (65·7%) (47·8–80·9)	4 (11·4%) (3·2–26·7%)
	Dose 2	10 (28·6%) (14·6–46·3)	14 (42·4%) (25·5–60·8)	16 (47·1%) (29·8–64·9)	20 (58·8%) (40·7–75·4)	24 (68·6%) (50·7–83·1)	10 (29·4%) (15·1–47·5)
Pain	Dose 1	2 (5·7%) (0·7–19·2)	8 (22·9%) (10·4–40·1)	10 (28·6%) (14·6–46·3)	16 (45·7%) (28·8–63·4)	16 (45·7%) (28·8–63·4)	3 (8·6%) (1·8–23·1)
	Dose 2	10 (28·6%) (14·6–46·3)	10 (28·6%) (14·6–46·3)	15 (42·9%) (26·3–60·6)	17 (48·6%) (31·4–66·0)	22 (62·9%) (44·9–78·5)	8 (22·9%) (10·4–40·1)
Tenderness	Dose 1	6 (17·1%) (6·6–33·6)	4 (11·4%) (3·2–26·7)	4 (11·4%) (3·2–26·7)	4 (11·4%) (3·2–26·7)	7 (20·0%) (8·4–36·9)	1 (2·9%) (0·1–14·9)
	Dose 2	0 (0·0%) (0·0–10·0)	4 (11·4%) (3·2–26·7)	1 (2·9%) (0·1–14·9)	3 (8·6%) (1·8–23·1)	2 (5·7%) (0·7–19·2)	2 (5·7%) (0·7–19·2)
Swelling	Dose 1	0 (0·0%) (0·0–10·0)	0 (0·0%) (0·0–10·0)	0 (0·0%) (0·0–10·0)	0 (0·0%) (0·0–10·0)	0 (0·0%) (0·0–10·0)	0 (0·0%) (0·0–10·0)
	Dose 2*	masked	masked	masked	masked	masked	masked
Induration	Dose 1	0 (0·0%) (0·0–10·0)	0 (0·0%) (0·0–10·0)	0 (0·0%) (0·0–10·0)	0 (0·0%) (0·0–10·0)	0 (0·0%) (0·0–10·0)	0 (0·0%) (0·0–10·0)
	Dose 2	0 (0·0%) (0·0–10·0)	0 (0·0%) (0·0–10·0)	0 (0·0%) (0·0–10·0)	0 (0·0%) (0·0–10·0)	0 (0·0%) (0·0–10·0)	0 (0·0%) (0·0–10·0)
Erythema	Dose 1	0 (0·0%) (0·0–10·0)	0 (0·0%) (0·0–10·0)	0 (0·0%) (0·0–10·0)	0 (0·0%) (0·0–10·0)	0 (0·0%) (0·0–10·0)	0 (0·0%) (0·0–10·0)
	Dose 2	0 (0·0%) (0·0–10·0)	0 (0·0%) (0·0–10·0)	0 (0·0%) (0·0–10·0)	0 (0·0%) (0·0–10·0)	0 (0·0%) (0·0–10·0)	0 (0·0%) (0·0–10·0)
Any systemic AE	Dose 1	12 (34·3%) (19·1–52·2)	8 (22·9%) (10·4–40·1)	19 (54·3%) (36·6–71·2)	14 (40·0%) (23·9–57·9)	17 (48·6%) (31·4–66·0)	7 (20·0%) (8·4–36·9)
	Dose 2	9 (25·7%) (12·5–43·3)	11 (33·3%) (18·0–51·8)	9 (26·5%) (12·9–44·4)	17 (50·0%) (32·4–67·6)	15 (42·9%) (26·3–60·6)	4 (11·8%) (3·3–2·.5)
Fever >38 °C	Dose 1	0 (0·0%) (0·0–10·0)	0 (0·0%) (0·0–10·0)	1 (2·9%) (0·1–14·9)	0 (0·0%) (0·0–10·0)	3 (8·6%) (1·8–23·1)	0 (0·0%) (0·0–10·0)
	Dose 2	0 (0·0%) (0·0–10·0)	0 (0·0%) (0·0–10·0)	1 (2·9%) (0·1–14·9)	2 (5·7%) (0·7–19·2)	2 (5·7%) (0·7–19·2)	0 (0·0%) (0·0–10·0)
Headache	Dose 1	5 (14·3%) (4·8–30·3)	5 (14·3%) (4·8–30·3)	9 (25·7%) (12·5–43·3)	6 (17·1%) (6·6–33·6)	11 (31·4%) (16·9–49·3)	1 (2·9%) (0·1–14·9)
	Dose 2	4 (11·4%) (3·2–26·7)	5 (14·3%) (4·8–30·3)	6 (17·1%) (6·6–33·6)	8 (22·9%) (10·4–40·1)	4 (11·4%) (3·2–26·7)	1 (2·9%) (0·1–14·9)
Fatigue	Dose 1	8 (22·9%) (10·4–40·1)	4 (11·4%) (3·2–26·7)	12 (34·3%) (19·1–52·2)	6 (17·1%) (6·6–33·6)	6 (17·1%) (6·6–33·6)	7 (20·0%) (8·4–36·9)
	Dose 2	3 (8·6%) (1·8–23·1)	6 (17·1%) (6·6–33·6)	7 (20·0%) (8·4–36·9)	8 (22·9%) (10·4–40·1)	7 (20·0%) (8·4–36·9)	4 (11·4%) (3·2–26·7)
Malaise	Dose 1	1 (2·9%) (0·1–14·9)	1 (2·9%) (0·1–14·9)	1 (2·9%) (0·1–14·9)	3 (8·6%) (1·8–23·1)	4 (11·4%) (3·2–26·7)	0 (0·0%) (0·0–10·0)
	Dose 2	1 (2·9%) (0·1–14·9)	1 (2·9%) (0·1–14·9)	1 (2·9%) (0·1–14·9)	4 (11·4%) (3·2–26·7)	4 (11·4%) (3·2–26·7)	0 (0·0%) (0·0–10·0)
Myalgia	Dose 1	4 (11·4%) (3·2–26·7)	4 (11·4%) (3·2–26·7)	8 (22·9%) (10·4–40·1)	6 (17·1%) (6·6–33·6)	9 (25·7%) (12·5–43·3)	1 (2·9%) (0·1–14·9)
	Dose 2	6 (17·1%) (6·6–33·6)	2 (5·7%) (0·7–19·2)	4 (11·4%) (3·2–26·7)	11 (31·4%) (16·9–49·3)	11 (31·4%) (16·9–49·3)	1 (2·9%) (0·1–14·9)
Arthralgia	Dose 1	2 (5·7%) (0·7–19·2)	1 (2·9%) (0·1–14·9)	5 (14·3%) (4·8–30·3)	0 (0·0%) (0·0–10·0)	3 (8·6%) (1·8–23·1)	0 (0·0%) (0·0–10·0)
	Dose 2	2 (5·7%) (0·7–19·2)	1 (2·9%) (0·1–14·9)	2 (5·7%) (0·7–19·2)	4 (11·4%) (3·2–26·7)	4 (11·4%) (3·2–26·7)	0 (0·0%) (0·0–10·0)
Nausea	Dose 1	2 (5·7%) (0·7–19·2)	1 (2·9%) (0·1–14·9)	3 (8·6%) (1·8–23·1)	1 (2·9%) (0·1–14·9)	1 (2·9%) (0·1–14·9)	0 (0·0%) (0·0–10·0)
	Dose 2	3 (8·6%) (1·8–23·1)	1 (2·9%) (0·1–14·9)	1 (2·9%) (0·1–14·9)	2 (5·7%) (0·7–19·2)	0 (0·0%) (0·0–10·0)	0 (0·0%) (0·0–10·0)
Vomiting	Dose 1	1 (2·9%) (0·1–14·9)	0 (0·0%) (0·0–10·0)	0 (0·0%) (0·0–10·0)	1 (2·9%) (0·1–14·9)	1 (2·9%) (0·1–14·9)	0 (0·0%) (0·0–10·0)
	Dose 2⁎⁎	masked	masked	masked	masked	masked	masked

⁎ Swelling after dose 2 was reported by 1 subject whose treatment group remains masked d (0·5%, 95% CI 0·0–2·6).

⁎⁎ Vomiting after dose 2 was reported by 1 subject whose treatment group remains masked (0·5%, 95% CI 0·0–2·6).

**Table 3:** AEs with onset during 28 days after vaccination.

		1 µg S(*N* = 35)	1 µg S+CpG(*N* = 35)	3 µg S(*N* = 35)	3 µg S+CpG(*N* = 35)	10 µg S(*N* = 35)	Placebo(*N* = 35)
		n (%)(95% CI)	n (%)(95% CI)	n (%)(95% CI)	n (%)(95% CI)	n (%)(95% CI)	n (%)(95% CI)
Any	Dose 1	8 (22·9%) (10·4–40·1)	7 (20·0%) (8·4–36·9)	15 (42·9%) (26·3–60·6)	13 (37·1%) (21·5–55·1)	9 (25·7%) (12·5–43·3)	6 (17·1%) (6·6–33·6)
	Dose 2	7 (20·0%) (8·4–36·9)	3 (8·6%) (1·8–23·1)	11 (31·4%) (16·9–49·3)	10 (28·6%) (14·6–46·3)	6 (17·1%) (6·6–33·6)	9 (25·7%) (12·5–43·3)
Vaccine-related	Dose 1	2 (5·7%) (0·7–19·2)	3 (8·6%) (1·8–23·1)	3 (8·6%) (1·8–23·1)	5 (14·3%) (4·8–30·3)	3 (8·6%) (1·8–23·1)	0 (0·0%) (0·0–10·0)
	Dose 2	0 (0·0%) (0·0–10·0)	1 (2·9%) (0·1–14·9)	2 (5·7%) (0·7–19·2)	2 (5·7%) (0·7–19·2)	2 (5·7%) (0·7–19·2)	1 (2·9%) (0·1–14·9)
Serious	Dose 1*	masked	masked	masked	masked	masked	masked
	Dose 2	0 (0·0%) (0·0–10·0)	0 (0·0%) (0·0–10·0)	1⁎⁎ (2·9%) (0·1–14·9)	0 (0·0%) (0·0–10·0)	1⁎⁎ (2·9%) (0·1–14·9)	1⁎⁎ (2·9%) (0·1–14·9)
Serious vaccine-related	Dose 1	0 (0·0%) (0·0–10·0)	0 (0·0%) (0·0–10·0)	0 (0·0%) (0·0–10·0)	0 (0·0%) (0·0–10·0)	0 (0·0%) (0·0–10·0)	0 (0·0%) (0·0–10·0)
	Dose 1	0 (0·0%) (0·0–10·0)	0 (0·0%) (0·0–10·0)	0 (0·0%) (0·0–10·0)	0 (0·0%) (0·0–10·0)	0 (0·0%) (0·0–10·0)	0 (0·0%) (0·0–10·0)

⁎ Serious AE (abrasion wound from motorcycle accident) was reported by one subject whose treatment group remains masked (0·5%, 95% CI 0·0–2·6).

⁎⁎ Serious AEs (asymptomatic SARS-CoV-2 infection in 3 µg S and placebo groups, tramadol overdose in 10 µg S group.

**Figure 2:**
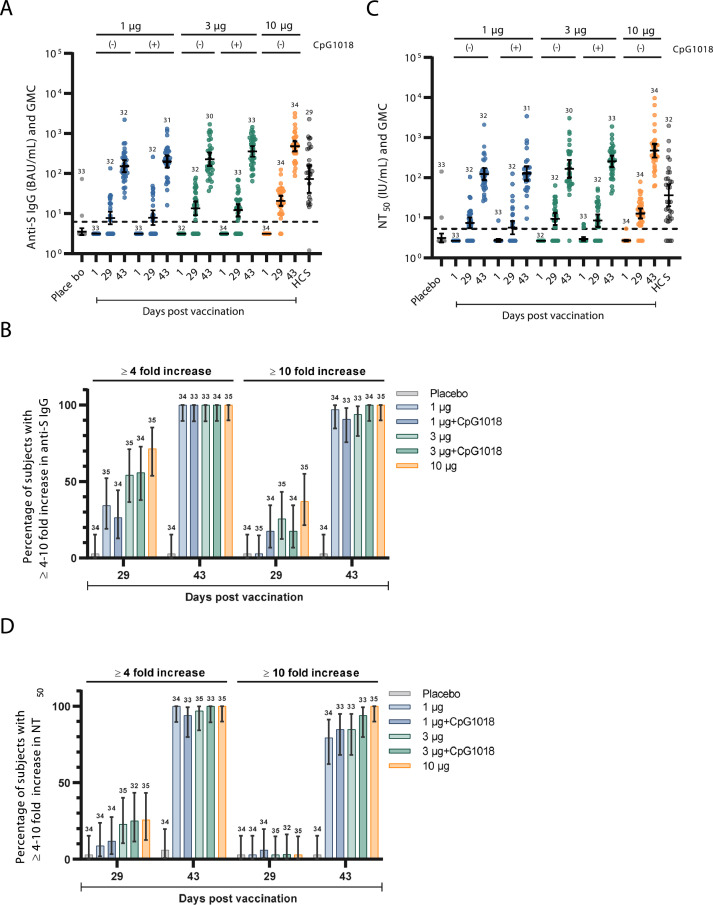
Humoral immune responses to five NDV-HXP-S vaccine formulations in vaccinated subjects and placebo controls measured at baseline (day 1), post dose 1 (day 29) and post dose 2 (day 43), contrasted to a reference panel of human convalescent sera (HCS). (A) Distribution and GMC of anti-S IgG by ELISA (BAU/mL) and (B) percentage of subjects with ≥ 4-fold and ≥ 10-fold increase; (C) distribution and GMC of NT_50_ by pseudotyped virus neutralization assay (IU/mL) and (D) percentage of subjects with ≥ 4-fold and ≥ 10-fold increase. Numbers above data denote number of per-protocol subjects contributing data; the central horizonal bar denotes the geometric mean, while error bars denote the 95% CI of the mean (A, C) or of the percentage (B, D).

Two doses of NDV-HXP-S were immunogenic in a formulation and dose dependant manner within the per protocol population. Induction of anti-S IgG was modest following dose one but there was a marked anamnestic response observed 14 days after vaccine dose two (Figure 2A). Seronegative individuals in the vaccine groups responded 28 days after first vaccination with GMCs of anti-S IgG between 7·79 (1 µg) and 20·93 (10 µg) BAU/mL, with a ≥ 4-fold increase in 34·3–71·4%. The second dose considerably increased anti-S IgG antibody responses after 14 days to GMCs between 151·78 (1 µg) and 479·83 (10 µg) BAU/mL. All individuals in every vaccine group had a ≥ 4-fold increase over baseline after the second dose; all individuals in the 10 µg and 3 ug+CpG1018 groups had a ≥ 10-fold increase, as did > 90% of vaccinees in the other three vaccine groups (Figure 2B). Notably, the adjuvant effect of CpG was limited after two vaccine doses (Table 4): the 1 µg group had a GMC of 151·78 BAU/mL (95% CI 108·99–211·37) while the 1 µg+CpG1018 group had a GMC of 199·08 BAU/mL (95% CI 140·25–282·57). among recipients of the 3 µg dose, the GMC group difference appeared to be greater: 228·07 BAU/mL (no adjuvant, 95% CI 154·22–337·27) in contrast to 356·83 BAU/mL (CpG1018, 95% CI (265·89–478·88).Importantly, GMCs of anti-S IgG among the vaccine groups on day 43 exceeded the GMC of the HCS panel (*N* = 29, 72·93 BAU/mL, 95% CI 33.00–161.14) by 2–6-fold (Table 4).

**Table 4:** GMCs of anti-S IgG (BAU/mL) and NT_50_ by PNA (IU/mL) on day 43 and GMC ratios, vaccine to HCS panel.

		1 µg S	1 µg S+CpG	3 µg S	3 µg S+CpG	10 µg S
Anti-S IgG BAU/mL,	GMC	151·78	199·08	228·07	356·83	479·83
	95% CI	(108·99–211·37)	(140·25–282·57)	(154·22–337·27)	(265·89–478·88)	(360·19–639·20)
	GMC ratio, vaccine to HCS panel	2·08	2·73	3·13	4·89	6·58
	95% CI	(0·89–4·87)	(1·16–6·43)	(1.31–7·48)	(2·12–11·31)	(2·85–15·18)
NT50 IU/mL by PNA	GMC	122·23	127·92	166·54	257·70	474·35
	95% CI	(86·40–172·91)	(85·08–192·34)	(100·19–276·81)	(187·01–355·11)	(320·90–701·19)
	GMC ratio, vaccine to HCS panel	3·37	3·52	4·59	7·10	13·07
	95% CI	(1·67–6·81)	(1·69–7·34)	(2·08–10·10)	(3·55–14·20)	(6·33–26·99)

Functional antibody responses were assessed by PNA. Low NT_50_ GMCs were detected in all vaccine groups after the first vaccination (between 7·49 and 12·82 IU/mL) with ≥ 4-fold rises in 8·8% to 25·7% of the vaccine groups (Figure 2C, D). The second vaccine dose strongly boosted neutralisation GMCs to between 122·23 IU/mL (1 µg, 95% CI 86·40–172·91) and 474·35 IU/mL (10 µg, 95% CI 320·90–701·19), with a ≥ 4-fold increase over baseline in 93·9% to 100% of vaccine groups and a ≥ 10-fold rise in most individuals (100% in the 10 µg group, and between 79·4% and 93·9% in the remaining groups). The differences in post-second dose neutralising antibody GMCs between the unadjuvanted and adjuvanted 1 µg and 3 µg groups were absent or modest, respectively: 1 µg, 122·23 IU/mL (95% CI 86·40–172·91) versus 1 µg + CpG1018, 127·92 IU/mL (95% CI 85·08–192·34); 3 µg, 166·54 IU/m: (95% CI 100·19–276·81) versus 3 µg+CpG1018 257·70 IU/mL (95% CI 187·01–355·11).

Based on the vaccine-homologous binding and neutralising antibody responses, there was a clear ranking of immunogenicity with the 10 µg formulation performing best followed by the 3 µg+CpG1018, 3 µg, 1 µg+CpG1018 and 1 µg formulations. The induction of humoral immunity was strong with post-boost GMFRs relative to baseline of 48-fold (1 µg) to 152-fold (10 µg) for anti-S IgG and 46-fold (1 µg) to 174-fold (10 µg) for NT_50_ antibodies (Fig. S1). Notably, GMCs of NT_50_ by PNA among the vaccine groups on day 43 exceeded the GMC of the HCS panel (*N* = 32, 36·30 IU/mL, 95% CI 19·43–67·79) by 3–13-fold depending on the vaccine formulation (Table 4).

**Figure 3:**
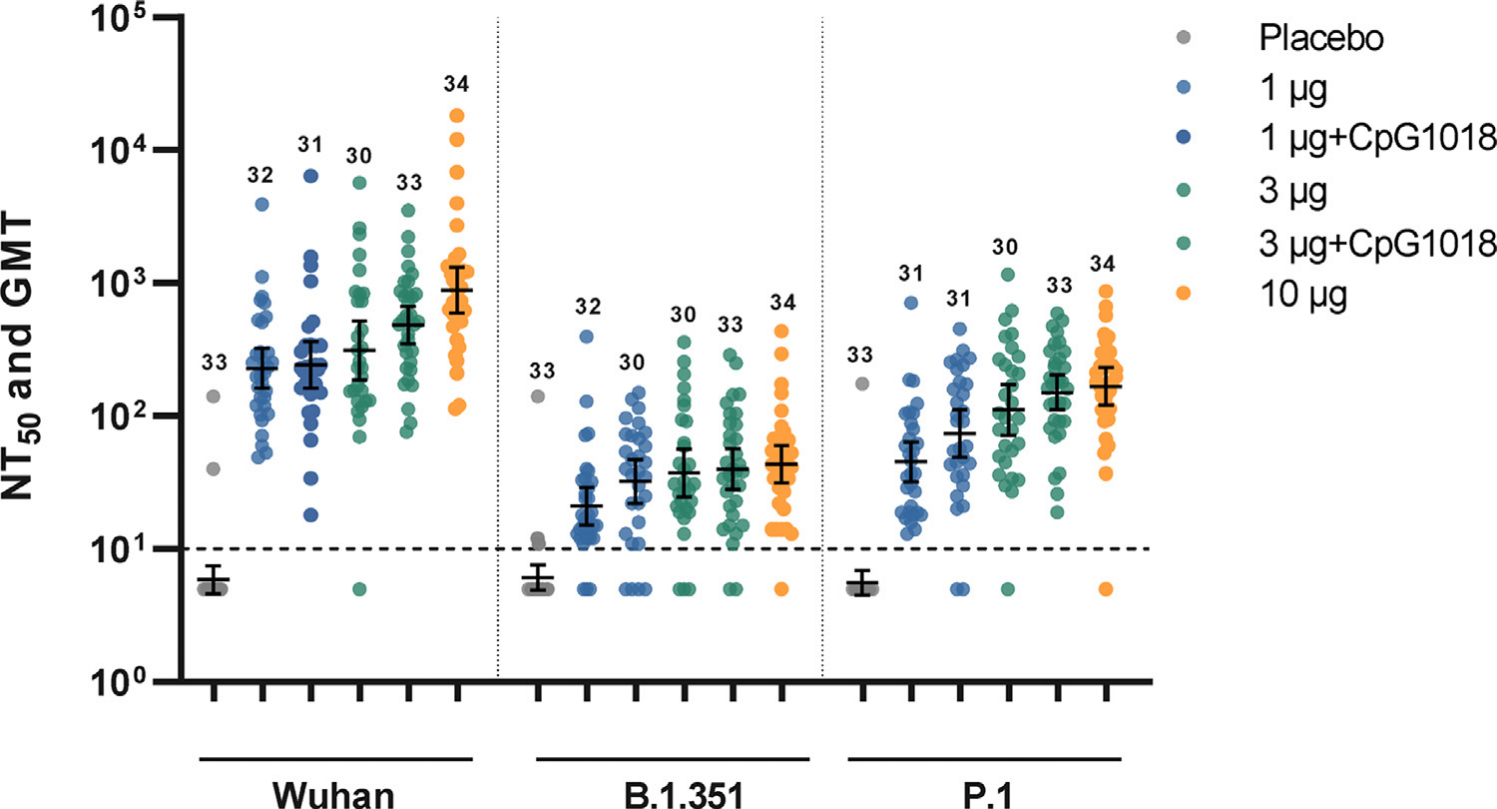
Distribution of neutralizing antibody responses (NT_50_) against vaccine homologous pseudotyped virus (Wuhan-Hu-1) and pseudotyped variants of concern B.1.351 and P.1 measured post-dose 2 (day 43), comparing five NDV-HXP-S vaccine formulations in placebo controls and vaccinated subjects, Numbers above data denote the number of per-protocol subjects contributing data; geometric mean and 95%CI are shown by horizontal bars.

**Figure 4:**
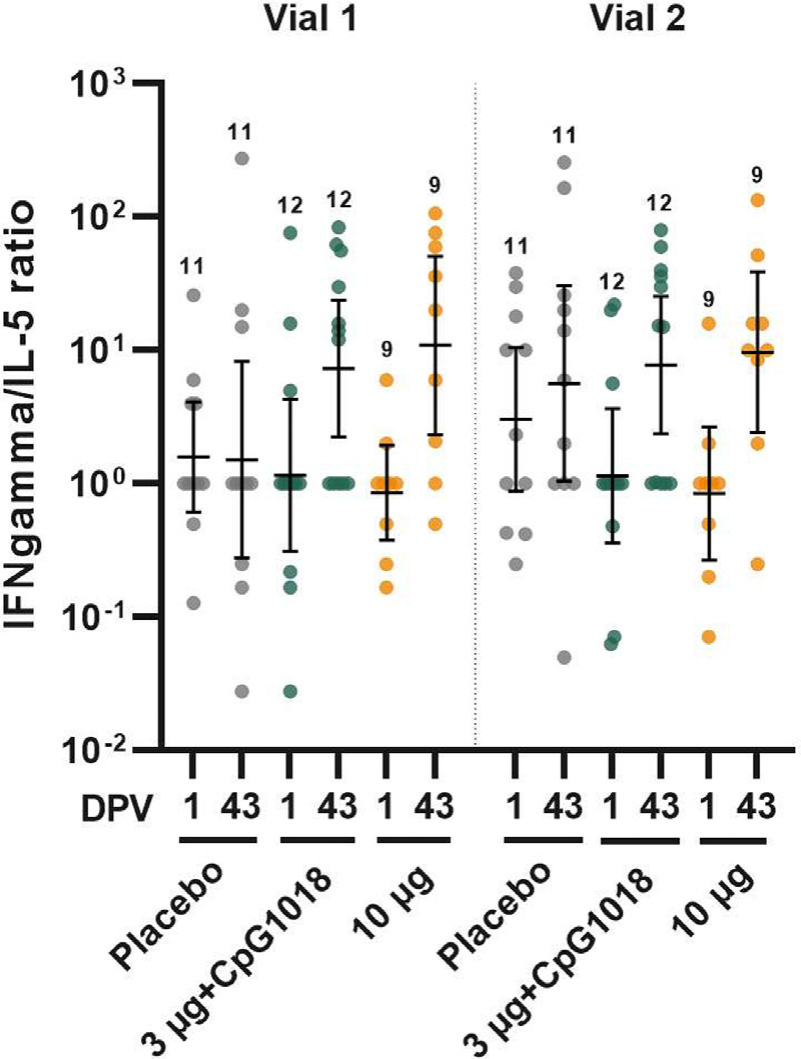
Induction of cell-mediated immunity by two NDV-HXP-S formulations versus placebo control, expressed as IFN-γ/IL-5 ratios determined by ELISpot 2-colour assay of PBMCs collected at baseline (day 1) and 14 days post-dose 2 (day 43). PBMCs were stimulated with peptides spanning the full-length S protein, in two separate but concurrent assays labelled Vial 1 and Vial 2. Numbers above data denote number of per-protocol subjects contributing data; horizontal bars denote the geometric mean ratio with 95% CI.

Additionally, neutralisation of variant viruses was assessed by PNA on day 43; this was pre-specified for B.1.351 and P.1 and thus tested for all dose groups; whilst the testing against B.1.617.2 was *post hoc* and done only for the 3 µg dose group. The proportion of subjects attaining a day 43 NT_50_ titre ≥40 increased with higher doses of antigen but the incremental changes in GMT were small (Figure 3 and Table 5). Reduction in post-dose 2 GMT of neutralising activity in subjects administered the 3 µg dose, relative to anti-Wuhan neutralising activity, was 2·8-fold for P.1 (95% CI 1·91–4·1), 3·32-fold for B.1.617.2 (95% CI 2·16–5·09), and 8.33-fold for B·1·351 (95% CI 5.38–12.9). In the 3 µg formulation groups, the proportion of 3 µg recipients attaining a NT_50_ titre ≥40 was 80% against P.1, 69% against B.1.617.2, and 43·3% against B.1.351 (Table 5). Finally, we also explored T cell responses to determine if the vaccine induced primarily a type 1 (T_H_1) or type 2 (T_H_2) T-helper cell response. In the small subset of subjects evaluated 14 days after a second dose, the IFN-γ/IL-5 ratio was strongly skewed to a T_H_1 response relative to pre-vaccination baseline (Figure 4), suggesting the vaccine induced T cell memory capable of an antiviral response.

**Table 5:** GMT and percentage of subjects with a titre ≥40 on day 43 for NT_50_ by PNA against Wuhan strain and three variants of concern.

		1 µg S	1 µg S+CpG	3 µg S	3 µg S+CpG	10 µg S	Placebo
Wuhan	GMT	*n* = 32 228·81 (161·74–323·69)	*n* = 31 239·47 (159·27–360·06)	*n* = 30 311·76 (187·56–518·18)	*n* = 33 482·42 (350·08–664·77)	*n* = 34 887·99 (600·72–1312·62)	*n* = 33 5·89 (4·64–7·48)
	titre ≥40	*N* = 34 34 (100%) (89·7–100)	*N* = 33 31 (93·9%) (79·8–99·3)	*N* = 33 32 (97·0%) (84·2–99·9)	*N* = 33 33 (100%) (89·4–100)	*N* = 35 35 (100%) (90·0–100)	*N* = 34 2 (5·9%) (0·7–19·7)
P·1	GMT	*n* = 31 45·33 (32·18–63·85)	*n* = 31 74·06 (48·99–111·97)	*n* = 30 111·28 (72·06–171·83)	*n* = 33 150·59 (111·15–204·03)	*n* = 34 167·14 (120·63–231·58)	*n* = 33 5·57 (4·47–6·94)
	titre ≥40	*N* = 31 15 (48·4%) (30·2 – 66·9)	*N* = 31 29 (74·2%) (55/4 – 88·1)	*N* = 30 24 (80·0%) (61·4 – 92·3)	*N* = 33 29 (87·9%) (71·8 – 96·6)	*N* = 34 (32 (94·9%) (80·3 – 99·3)	*N* = 33 1 (3·0%) (0·1 – 5·8)
B·1·351	GMT	*n* = 32 21·00 (15·12–29·18)	*n* = 30 32·34 (22·13 - 47·24)	*n* = 30 37·43 (24·77 - 56·56)	*n* = 33 40·07 (28·09 −57·17)	*n* = 34 43·47 (31·55 - 59·89)	*n* = 33 6·12 (4·90 - 7·64)
	titre ≥40	*N* = 32 5 (15·6%) (5·3 – 32·8)	*N* = 30 15 (50·0%) (31·3 −68·7)	*N* = 30 13 (43·3%) (30·8 - 66·5)	*N* = 33 16 (48·5%) (30·8 – 66·5)	*N* = 34 20 (58·8%) (40·7 – 75·4)	*N* = 33 1 (3·0%) (0·1 – 15·8)
B.1.617.2	GMT	N.D.	N.D.	*n* = 29 95·07 (59·64–151·52)	N.D.	N.D.	N.D.
	titre ≥40	N.D.	N.D.	*N* = 29 20 (69·0%) (49·2–84.7)	N.D.	N.D.	N.D.

N.D. = not done.

## Discussion

Current production capacity cannot satisfy the global demand for COVID-19 vaccines^1^ and vaccine distribution is inequitable with most vaccines acquired and used by high income countries while LMICs have limited access. Furthermore, vaccines requiring very low temperature storage, such as messenger ribonucleic acid (mRNA)-based COVID-19 vaccines, may be ill suited programmatically for LMIC use. Thus, local production of COVID-19 vaccines compatible with prolonged 2–8 °C storage in LMICs would increase global availability and reduce dependence of countries producing these vaccines on international vaccine supply. Here we demonstrated for the first time that an engineered inactivated NDV-based vaccine expressing a second-generation (6-proline) stabilized SARS-CoV-2 S protein,^5^ produced in eggs in an existing influenza virus production facility at GPO in Thailand, shows an acceptable reactogenicity and safety profile in humans and has immunogenicity that suggests its potential clinical benefit. We evaluated a range of vaccine doses (1 µg, 3 µg, 10 µg) having potency quantified as µg of virus envelope-anchored SARS-CoV-2 S protein; the low and medium antigen doses were evaluated in formulations with and without the TLR-9 agonist CpG1018 as a vaccine adjuvant. Over 28 days after each vaccine dose, all formulations were very well-tolerated with little reactogenicity aside from mild injection site pain and tenderness. No clinically important treatment-related adverse event occurred during the 56 days of observation following first vaccination with any formulation. Moreover, the vaccine was strongly immunogenic in a formulation and dose dependant manner, inducing levels of vaccine-homologous anti-S IgG and virus neutralising antibodies that exceeded by several fold the levels measured in 14-day convalescent sera from consecutive cases of health care workers with mild to moderate COVID-19 illness in 2020. Unexpectedly, the value of adding the CpG1018 adjuvant to the low- and mid-dose formulations, as measured by enhanced induction of humoral immunity, was modest. On the other hand, the small sample size limited the precision of our estimates; moreover, elderly subjects, for whom an adjuvant effect may be more apparent, were not enroled in this study. Importantly, the vaccine at a 3 µg dose level elicited neutralising antibodies against three variants of concern: P.1, B.1.617.2, and B.1.351. While neutralising antibody titres decreased modestly against P.1 and B.1.617.2 and more markedly against B.1.351, this was expected and in the range observed with sera from recipients of the mRNA vaccines BNT162b2 and mRNA-1273.^14^, ^15^, ^16^, ^17^ The degree of reduction in neutralisation is dependant on the assay used and can be especially dramatic with pseudotyped particle inhibition assays as used in this study.^14^^,^^17^ The T cell response assessed showed a bias towards a T_H_1 response in both evaluated dose groups, alleviating concerns about enhanced disease associated with a T_H_2 response (as observed with SARS-CoV-1 in some animal models^18^). These initial data, while sparse, suggest the vaccine-induced T cell memory capable of an antiviral response.

The study has several limitations. The sample size per treatment group was small, reducing precision, and assessments were restricted to 43 days for immunogenicity and 57 days for reactogenicity and safety, narrowing our perspective to acute outcomes only. These are inherent problems of phase 1 trials and interim analyses in a pandemic response setting. Nevertheless, as clinical trials with similar vaccines are underway in Vietnam (NCT04830800) and Brazil (NCT04993209), we determined that publication of early data is a priority. The study had strengths as well. The vaccine construct is a novel platform expressing a second-generation pre-fusion stabilized S protein in a membrane-bound trimeric conformation. We hypothesize that these characteristics contribute to the vaccine's notable immunogenicity, particularly induction of virus-neutralizing activity, even without the CpG1018 adjuvant. The anti-S IgG ELISA and PNA used to assess vaccine-homologous NT_50_ activity were validated and results are expressed in International Units^9^ for future comparisons.

In this trial, the induction of anti-S binding and neutralising antibodies was contrasted with mean levels in human convalescent serum and found to be superior, especially in the mid- and high-dose groups. Correlation between neutralising antibody titres and vaccine efficacy recently has been shown,^19^, ^20^, ^21^ including individual protection as assessed by pseudotyped virus neutralisation.^21^ We observed that the ratio of anti-Wuhan strain pseudotyped virus neutralising activity post vaccination to post-infection was ≥ 3, a level that Earle and others suggested may be associated with high vaccine efficacy as reported for Pfizer and Moderna mRNA vaccines.^19^ Subsequent trials of the NDV-HXP-S vaccine candidate will contrast its immunogenicity to an authorised comparator vaccine to generate relative immunogenicity evidence that may be predictive of clinical benefit and support authorisation for emergency use. Once the vaccine is widely deployed under such an authorisation, its effectiveness can be confirmed in observational studies.

In summary, we have shown preliminarily that the inactivated NDV-HXP-S vaccine candidate has an acceptable safety profile and is highly immunogenic. This vaccine can be produced at low cost in any facility designed for production of inactivated influenza virus vaccine; such facilities are present in a number of LMICs.^2^ The high dose formulation was most immunogenic (NT_50_ GMC 13-fold higher than the HCS panel); however, the mid dose formulations without and with CpG1018 adjuvant also induced NT_50_ GMCs that exceeded the HCS panel GMC by 4- to 7-fold. Based on these relatively high neutralising antibody ratios and acknowledging the importance to maximise supply of vaccine doses from the manufacturing facility to fulfil its public sector mission, the 3 µg and 3 µg+CpG1018 formulations were selected for further assessment in the phase 2 stage of the ongoing clinical trial.

## Declaration of interests

PN, SSur, SPra, SPuk, RK, RSin, NS, SThe, TV, KP, and PW are salaried employees of the Government Pharmaceutical Organization (Thailand). RH is a paid consultant to PATH. WS reports royalty payments from Avimex. AGS reports financial support from the U.S. NIAID (Centers of Excellence for Influenza Research and Response 75N93021C00014, Collaborative Influenza Vaccine Innovation Centers contract 75N93019C00051). The AGS laboratory has received research support from Pfizer, Senhwa Biosciences, Kenall Manufacturing, Avimex, Johnson & Johnson, Dynavax, 7Hills Pharma, Pharmamar, ImmunityBio, Accurius, Nanocomposix, Hexamer, N-fold LLC, Model Medicines, and Merck. AGS has consulting agreements for the following companies involving cash and/or stock: Vivaldi Biosciences, Contrafect, 7Hills Pharma, Avimex, Vaxalto, Pagoda, Accurius, Esperovax, Farmak, Applied Biological Laboratories, Curelab Oncology, Curelab Veterinary, and Pfizer; he also has received royalties (Merck, Astrazeneca, BI Vetmedica, Avimex, Regeneron), payment for lectures (Seqirus, Pharmamar), and participates in scientific advisory boards for New York State on Covid-19 vaccines, Accurius, Vaxalto, and Pfizer PP reports financial support from the U.S. NIAID (Centers of Excellence for Influenza Research and Response 75N93021C00014, P01 AI097092–07, R01 AI145870–03, Collaborative Influenza Vaccine Innovation Centers contract 75N93019C00051), payments involving cash and/or stock from Avimex, Vaxalto, and Accurius, royalty payments from Astrazeneca, BI Vetmedica, and Avimex; he also reports participation on the following advisory boards: New York State advisory board on Covid-19 vaccines, Accurius, and Vaxalto. PP also reports philanthropic grants or contracts through Mount Sinai. FK reports financial support from the U.S. NIAID (Collaborative Influenza Vaccine Innovation Centers contract 75N93019C00051,  centre of Excellence for Influenza Research and Surveillance contract HHSN272201400008C), the JPB Foundation and the Open Philanthropy Project (research grant 2020–215,611, 5384), Pfizer, and the U.S. NCI ( contract 75N91019D00024, task order 75N91020F00003); he also has received royalties (Avimex), consulting fees (Pfizer, Seqirus, Third Rock Ventures, and Avimex), and payment for academic lectures during the past two years. NB reports participation as an AACR board member and support from SITC for travel and meeting attendance. CLH and JSM report financial support from the Bill & Melinda Gates Foundation and the U.S. NIH. The vaccine administered in this study was developed by faculty members at the Icahn School of Medicine at Mount Sinai including WS, PP, AGS, and FK. Mount Sinai has filed patent applications relating to SARS-CoV-2 serological assays and the NDV-based SARS-CoV-2 vaccine; the institution and its faculty inventors could benefit financially. JSM and CLH are inventors on a patent application concerning the Hexapro stabilized SARS-CoV-2 spike protein that was filed by the University of Texas at Austin and has been licensed to multiple entities; the university and its faculty inventors could benefit financially. All other authors have nothing to declare.

## Appendix Supplementary materials

Image, application 1

Image, application 2

## References

[1] (WHO) WHO. https://www.who.int/director-general/speeches/detail/director-general-s-opening-remarks-at-the-media-briefing-on-covid-19-9-april-2021. 2021.

[2] Sparrow E., Wood J.G., Chadwick C. (2021). Global production capacity of seasonal and pandemic influenza vaccines in 2019. Vaccine.

[3] Sun W., McCroskery S., Liu W.C. (2020). A Newcastle disease virus (NDV) expressing a membrane-anchored spike as a cost-effective inactivated SARS-CoV-2 vaccine. Vaccines (Basel).

[4] Hsieh C.L., Goldsmith J.A., Schaub J.M. (2020). Structure-based design of prefusion-stabilized SARS-CoV-2 spikes. Science.

[5] Sun W., Liu Y., Amanat F. (2021). A Newcastle disease virus- expressing a stabilized spike protein of SARS-CoV-2 induces protective immune responses. Nat Commun.

[6] Campbell J.D. (2017). Development of the CpG adjuvant 1018: a case study. Methods Mol Biol.

[7] ter Meulen J., van den Brink E.N., Poon L.L. (2006). Human monoclonal antibody combination against SARS coronavirus: synergy and coverage of escape mutants. PLoS Med.

[8] Hsieh C.L., Goldsmith J.A., Schaub J.M. (2020). Structure-based design of prefusion-stabilized SARS-CoV-2 spikes. Science.

[9] Kristiansen P.A., Page M., Bernasconi V. (2021). WHO international standard for anti-SARS-CoV-2 immunoglobulin. Lancet.

[10] Bewley K.R., Coombes N.S., Gagnon L. (2021). Quantification of SARS-CoV-2 . neutralising antibody by wild-type plaque reduction neutralisation, microneutralisation and pseudotyped virus neutralisation assays. Nat Protoc.

[11] Havranek K.E., Jimenez A.R., Acciani M.D. (2020). SARS-CoV-2 spike alterations enhance pseudoparticle titers and replication-competent VSV-SARS-CoV-2 virus. Viruses.

[12] Tegally H., Wilkinson E., Giovanetti M. (2021). Detection of a SARS-CoV-2 variant of concern in South Africa. Nature.

[13] World Health Organisation. Tracking SARS-CoV-2 variants. [Internet]. 2021 [cited 2021Dec12]. Available from: https://www.who.int/en/activities/tracking-SARS-CoV-2-variants/

[14] Garcia-Beltran W.F., Lam E.C., St Denis K. (2021). Multiple SARS-CoV-2 variants escape neutralisation by vaccine-induced humoral immunity. Cell.

[15] Carreño J.M., Alshammary H., Singh G. (2021). Evidence for retained spike-binding and neutralizing activity against emerging SARS-CoV-2 variants in serum of COVID-19 mRNA vaccine recipients. eBioMedicine.

[16] Edara V.V., Norwood C., Floyd K. (2021). Infection- and vaccine-induced antibody binding and neutralisation of the B.1.351 SARS-CoV-2 variant. Cell Host Microbe.

[17] Shen X., Tang H., Pajon R. (2021). Neutralisation of SARS-CoV-2 Variants B.1.429 and B.1.351. N Engl J Med.

[18] Krammer F. (2020). SARS-CoV-2 vaccines in development. Nature.

[19] Earle K.A., Ambrosino D.M., Fiore-Gartland A. (2021). Evidence for antibody as a protective correlate for COVID-19 vaccines. Vaccine.

[20] Khoury D.S., Cromer D., Reynaldi A. (2021). neutralising antibody levels are highly predictive of immune protection from symptomatic SARS-CoV-2 infection. Nat Med.

[21] Gilbert P.B., Montefiori D.C., McDermott A. (2021). Immune correlates analysis of the mRNA-1273 COVID-19 vaccine efficacy trial. Science.

