# Supplementary material for: Safety and immunogenicity of an inactivated recombinant Newcastle disease virus vaccine expressing SARS-CoV-2 spike: Interim results of a randomised, placebo-controlled, phase 1 trial
Source: eClinicalMedicine. 2022 Mar 8;45:101323. doi: 10.1016/j.eclinm.2022.101323 (PMC8903824; doi:10.1016/j.eclinm.2022.101323)
Supplement: Supplementary file 1 [file mmc1.docx]

**Supplementary Material:** Safety and Immunogenicity of an Inactivated Recombinant Newcastle Disease Virus Vaccine Expressing SARS-CoV-2 Spike: Interim Results of a Randomised, Placebo-Controlled, Phase 1 Trial

Figure S1. Humoral immune responses to five NDV-HXP-S vaccine formulations in vaccinated subjects and placebo controls measured as the fold rise from baseline (day 1) to post dose 1 (day 29) and post dose 2 (day 43). (A) Distribution and geometric mean fold rise of anti-S IgG and (B) dAlsoistribution and geometric mean fold rise of NT_50_ by PNA. Numbers above data denote the number of per-protocol subjects contributing data; the central horizonal bar denotes the geometric mean, while the error bars denote the 95% CI of the mean.

The current amendment of the phase 1/2 clinical trial protocol is provided as a separate pdf file: GPO NDV-HXP-S Phase 1_2 Protocol Version 7.0 dated 01 Sep 2021
