# Supplementary figures and images for: Safety and immunogenicity of an inactivated recombinant Newcastle disease virus vaccine expressing SARS-CoV-2 spike: Interim results of a randomised, placebo-controlled, phase 1 trial
Source: eClinicalMedicine. 2022 Mar 8;45:101323. doi: 10.1016/j.eclinm.2022.101323 (PMC8903824; doi:10.1016/j.eclinm.2022.101323)

Figure 2

**A**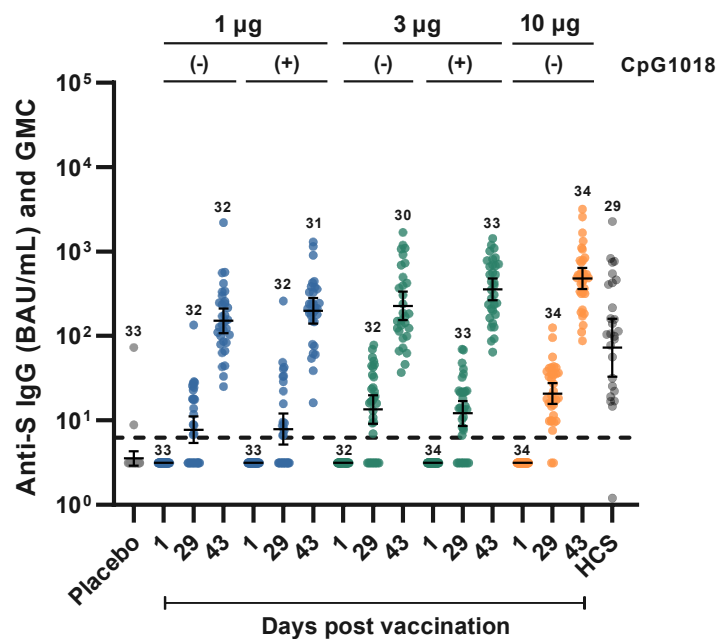**C**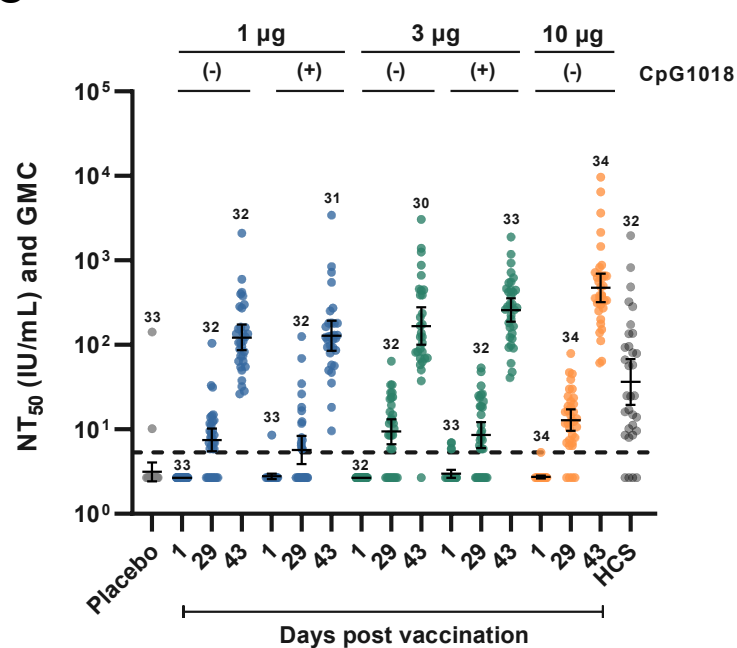**B**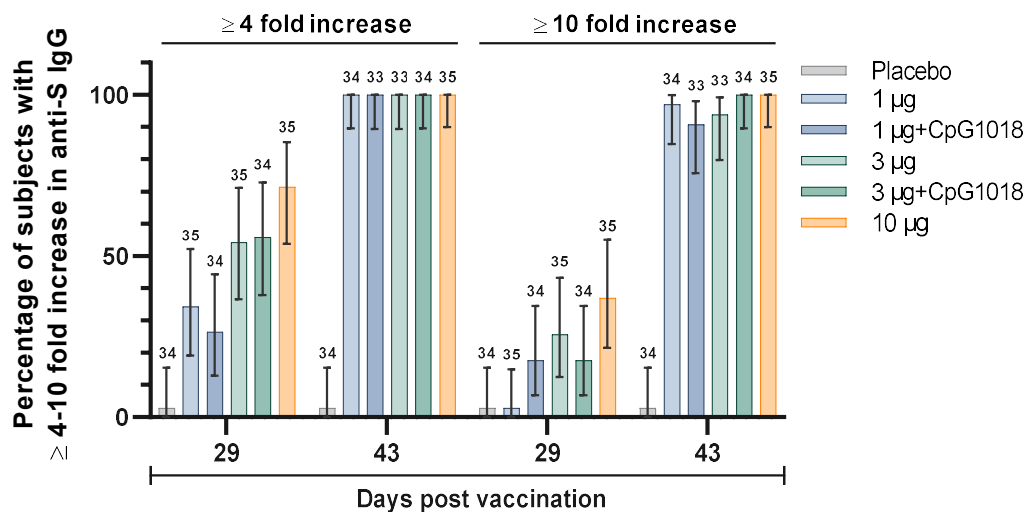**D**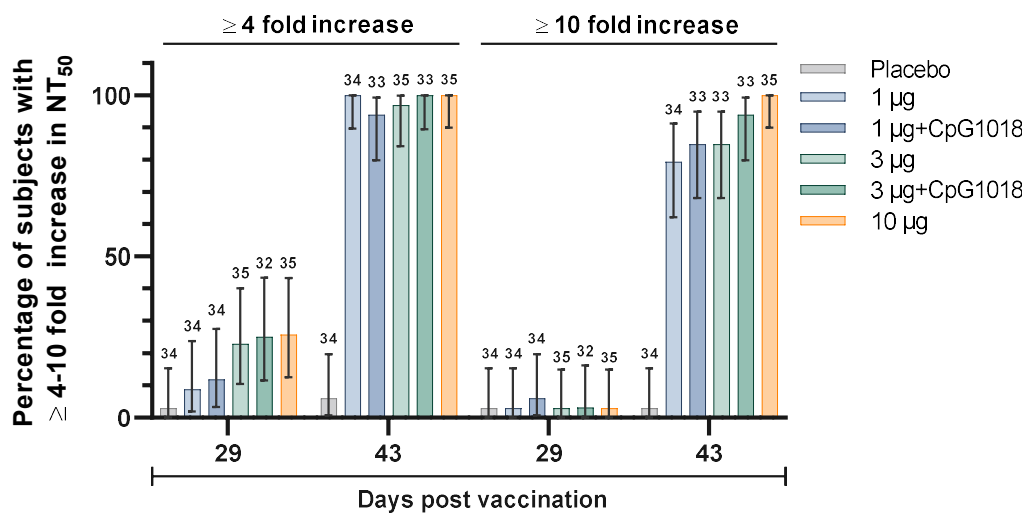

Figure 3

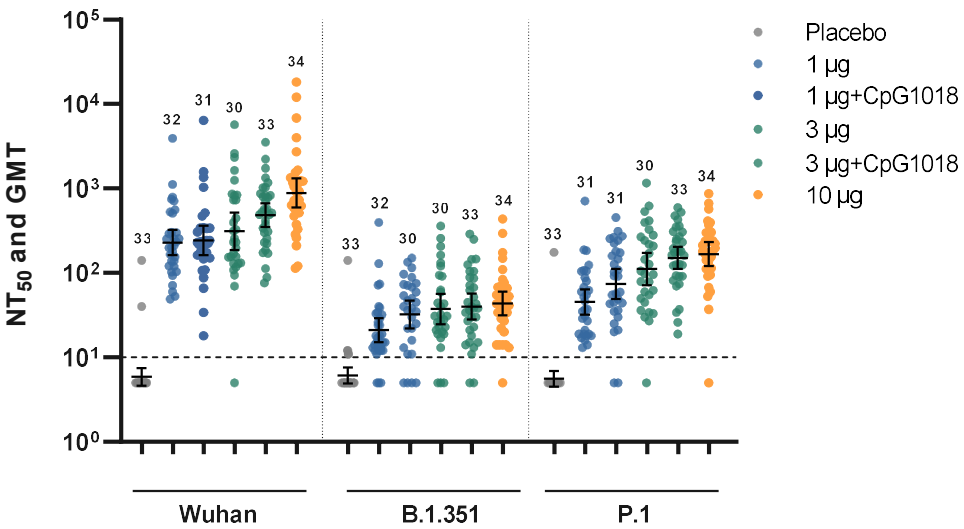

Figure 4

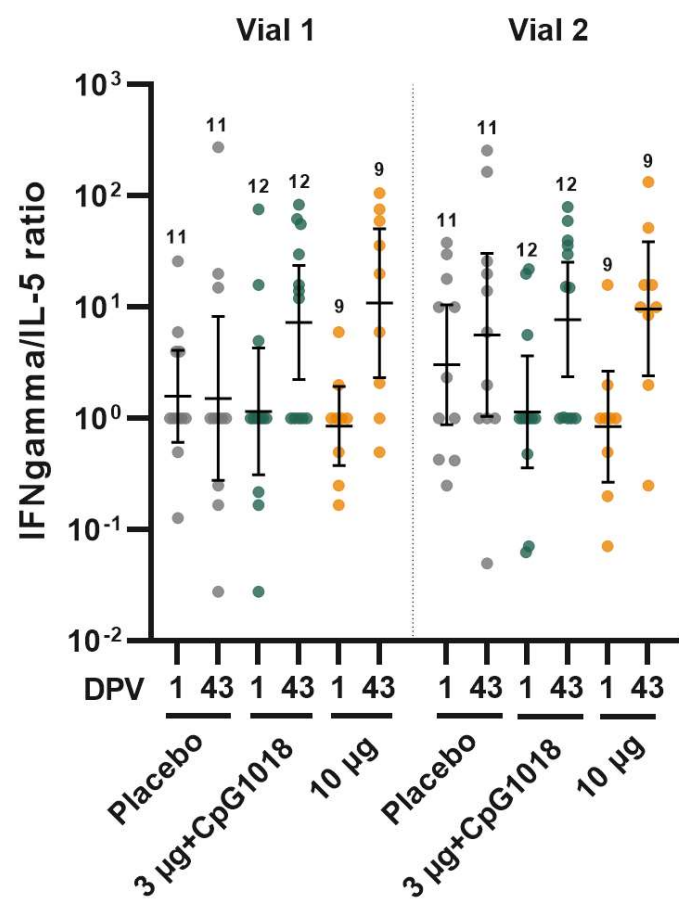

Figure S1

A

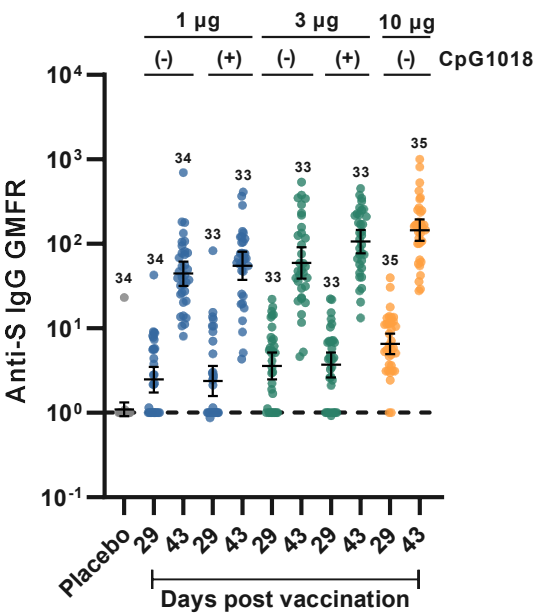

B

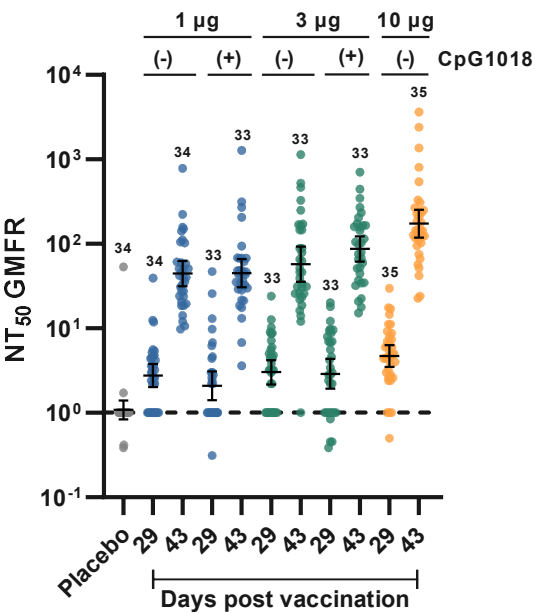

Supplement: Supplementary file 2 [file mmc2.pdf]
